# Effect of poloxamer 407 administration on the serum lipids profile, anxiety level and protease activity in the heart and liver of mice

**DOI:** 10.2478/intox-2013-0004

**Published:** 2013-03

**Authors:** Tatyana A. Korolenko, Thomas P. Johnston, Nina I. Dubrovina, Yana A. Kisarova, Svetlana Ya. Zhanaeva, Marina S. Cherkanova, Elena E. Filjushina, Tatyana V. Alexeenko, Eva Machova, Natalya A. Zhukova

**Affiliations:** 1 Institute of Physiology, Siberian Branch of Russian Academy of Medical Sciences, Novosibirsk, Russia; 2 University of Missouri-Kansas City, Kansas City, MO 64108, USA; 3 Institute of Chemistry, Slovak Academy of Sciences, Bratislava, Slovakia; 4 Voroztzov's Institute of Organic Chemistry, Siberian Branch of Russian Academy of Sciences, Novosibirsk, Russia

**Keywords:** Poloxamer 407, anxiety, serum lipids, cathepsins B, L, D, matrix metalloproteases

## Abstract

Chronic administration of the poloxamer 407 (P-407), a block copolymer, to elevate serum lipids in mice is a well-established mouse model of hyperlipidemia and atherosclerosis. We tested the hypothesis that the activity of several types of proteases in heart and liver tissue is changed in the early stages of atherosclerosis development. Additionally, we evaluated whether increased serum lipids would induce anxiety in mice, as determined by using a ‘plus-maze’ test. The mice were administered P-407 by intraperitoneal injection twice a week for one month. P-407 administration to mice resulted in a marked increase in total serum cholesterol, atherogenic non-HDL-cholesterol, and especially in total triglycerides, and it also increased anxiety. Morphological changes observed in P-407-treated mice included contractile type changes in cardiomyocytes and foamy macrophages in liver. A significant increase of cysteine proteases cathepsin B and cathepsin L (at 24 h) and aspartate protease cathepsin D (at both 24 h and 5 days) was determined in heart tissue following P-407 administration. However, no changes were noted in heart matrix metalloproteinase activity. The activity of cysteine and aspartate proteases was significantly increased in liver at both 24 hours and 5 days after P-407 administration. In conclusion, administration of P-407 to mice for one month resulted in increased anxiety, and more importantly, there was an increase in the activity of heart and liver proteases secondary to sustained dyslipidemia. It is suggested that heart and liver cysteine and aspartate proteases may represent potential therapeutic targets in the early stages of atherosclerosis.

## Introduction

Early diagnosis and reversibility of changes observed with atherosclerosis are important to clinical medicine and to the patients affected by this disease. Thus, it is not surprising that atherosclerosis and its many complications have been, and continue to be, intensively investigated using interdisciplinary approaches or strategies (Cullen *et al*., 2005). Recently, it was shown that protein degradation by different proteases is partly responsible for cardiovascular dysfunction in various types of heart diseases, including atherosclerosis (Brömme & Wilson, 2011). Some evidence was obtained supporting the hypothesis that the proteolytic enzyme activity of cathepsin B reflects the inflammatory component of atherosclerotic pathology and can quantitatively demonstrate the antiatherosclerotic therapeutic effects of statins (Kim *et al*., 2009). The emerging roles of cysteine proteases is related to their potential as drug targets (Vasiljeva *et al*., 2007). Matrix metalloproteases (MMP) have been shown to be involved in the pathogenesis in the late stage of atherosclerosis, especially during plaque formation and destabilisation of plaque (Chow *et al*., 2007; Muller *et al*., 2012). However, the involvement of different classes of proteases in the onset of *early* atherosclerosis in humans is still not known. This is one of the many reasons that experimental animal models, which can reliably recapitulate the phenotypic signatures or manifestations of human atherosclerosis, are so useful for investigating the dynamic processes associated with this disease state. It has been previously shown that repeated administration of the synthetic copolymer called poloxamer 407 (P-407) to mice resulted in dyslipidemia, and subsequently atherosclerosis (Johnston, 2009, 2010). Mice begin to form fibrofatty lesions in their aortas beginning 1 month after initiation of treatment (Johnston, 2004; Palmer *et al*., 1998). After 4 months of P-407 treatment, aortic atherosclerotic lesions are produced in the same size and density as those observed in classic diet-induced mouse models to induce atheroma formation (Johnston, 2010; Johnston *et al*., 2001). Recently, it was shown that repeated administration of P-407 to mice over a 4-month period induced atherosclerosis secondary to sustained dyslipidemia with significant changes in heart vessels. Specifically, repeated P-407 administration over 4 months resulted in damage to endothelial cells and increased activity of cysteine protease cathepsin B and matrix metalloprotease (MMP) in heart tissue, which was associated with cardiomyocyte injury (Korolenko *et al*., 2012b). Using a 1-month model of P-407 administration to mice, it is possible to investigate the *early* stages of atherosclerosis development, as well as their reversibility at the beginning of the pathological process. Among the manifestations involved in the pathogenesis of atherosclerosis, it is important to note early behavioral changes, such as anxiety level, depression, and movement activity, which are well known in human beings (Ohira *et al*., 2012).

Tissue proteases (cathepsins) show differential expression in various stages of atherosclerosis (Sjoberg & Shi, 2011), and *in vivo* knockout studies revealed that deficiency of cathepsin K or S reduced atherosclerosis (Bromme & Wilson, 2011). Furthermore, cathepsins are involved in lipid metabolism; they have the capability to degrade low-density lipoprotein and reduce cholesterol efflux from macrophages, aggravating foam cell formation (Bellosta & Bernini, 2005; Derosa *et al*., 2009).

Therefore, the aim of this study was to investigate the effects of repeated (over one month) P-407 treatment on the serum lipid profile, anxiety level, and the activity of several classes of proteases (cysteine, aspartate, MMP) involved in the *early* pathogenesis of atherosclerosis caused by sustained dyslipidemia.

## Materials and methods

Male CBA mice (breeding station of the Institute of Physiology, Siberian Branch of the Russian Academy of Medical Sciences, Novosibirsk, Russia), having a body mass of 25–30 g were used. All animal procedures were carried out in accordance with approved protocol and recommendations for proper use and care of laboratory animals (European Communities Council Directive 86/609/CEE). Poloxamer 407 (P-407) (Pluronic F-127, Sigma-Aldrich, St. Louis, MO, USA) was administered to mice, as an intraperitoneal (i.p.) injection at a dose of 500 mg/kg twice per week for 1 month according to Johnston (2004). The animals were euthanized 24 h after the last dose of P-407, when significant cholesterolemia and triglyceridemia were noted, and also at 5 days after the last dose of P-407 was administered. The latter time point (5 days post-treatment) was selected because the plasma concentrations of cholesterol and triglycerides have been demonstrated to return to baseline by this time after a single dose of P-407 (Johnston, 2009). Control mice received the equivalent volume of saline. The mice were deprived of food and had free access to water 15 h before euthanasia.

### Plus-maze method of behavioral analysis of mice

The elevated plus-maze test is probably the most popular of all currently available animal tests on anxiety, based on the study of unconditioned or spontaneous behavior (Rotgers & Dalvi, 1997). The elevated plus-maze apparatus (San Diego Instruments, San Diego, CA, USA) is comprised of two open arms (30 x 5 cm) and two enclosed arms (30 × 5 × 15 cm), which extend from a common central platform (5 × 5 cm). The configuration forms the shape of a plus-sign, with like arms arranged opposite one another, and the apparatus was elevated 60 cm above floor level on a central pedestal. Mice are placed on the central platform and for 5 min the conventional measurements comprised: time spent on the open arms, including the central platform, number of open arm entries, number of transitions from one enclosed arm to the other enclosed arm, number of glances from the enclosed arm, and level of defecation during the observation period.

### Serum

Serum was obtained after centrifugation of blood samples at 3000 × g for 20 min at 4 °C (Eppendorf Centrifuge 5415 R, Hamburg, Germany) and stored at –70 °C until analysis. In serum, the total cholesterol and TG concentrations were determined using Triglycerides-Novo and Novochol kits (Vector-Best, Novosibirsk Region, Russia). Serum HDL-cholesterol was assayed using a commercial kit called HDL-Cholesterol-Novo (Vector Best, Novosibirsk Region, Russia) and non-HDL-cholesterol was calculated by subtracting HDL-cholesterol from the total serum cholesterol. Photometry of the samples was performed on a Riele 5010 V5+ semiautomatic photometer (Robert Riele, Berlin, Germany) with a temperature-controlled flow-through cuvette.

### Cysteine proteases activity assay

Cathepsin B (EC 3.4.22.1) and cathepsin L (EC 3.4.22.15) specific activity in homogenates of liver and heart was measured according to a previously described method (Barrett and Kirschke, 1981), using the fluorogenic methylcoumarylamide substrates Z-Arg-Arg-MCA and Z-Phe-Arg-MCA (where Z = carbobenzoxy) (Sigma-Aldridge, St. Louis, MO, USA), 5 µM at pH 6.0 (cathepsin B, phosphate buffer, 0.2 M) and pH 5.5 (cathepsin L, acetate buffer, 0.2 M). Incubation was performed in the presence of 0.1% Triton X-100 to destroy the cellular membranes. Fluorescence measurements were recorded on a Shimadzu RF-5301PC spectrofluorophotometer (Tokyo, Japan) at 355 nm (excitation) and 460 nm (emission). 7-Amino-4-methylcoumarin (MCA, Sigma-Aldrich, St. Louis, MO, USA) served as a standard. The results were expressed as µmol of MCA cleaved per min per g of protein. In the cathepsin L assay, a specific inhibitor of cathepsin B (CA-074, 0.3 µmol) was added to the incubation medium to exclude the activity of cathepsin B. Protein concentration was assayed according to Lowry *et al*. (1951).

### Cathepsin D activity assay

Cathepsin D (EC 3.4.23.5) activity in homogenates of liver and heart was determined using 2% azocasein (Sigma-Aldrich, St. Louis, MO, USA) dissolved in 6 M urea (pH 5.0) as the substrate (Wiederanders et al., 1986). The cathepsin D activity was expressed as A366 per min per mg of protein (arbitrary units).

### Matrix metalloprotease activity assay

The activity of matrix metalloproteases (MMP) in liver, heart, and spleen homogenates was determined by a fluorescent method according to Knight *et al.* (1992) using the substrate MCA-Pro-Leu-Gly∼Leu-DpA-Ala-Arg-NH2 (American Peptide Co., Sunnyvale, CA, USA), 1.6 mM, at pH 7.5. To exclude the effect of serine proteases in the cleavage of this substrate, MMP activity was measured in the presence of an inhibitor of serine protease, phenylmethylsulphonyl fluoride (PMSF, Boehringer Mannheim, Mannheim, Germany), at a final concentration of 0.5 mM (Korolenko *et al.*, 2011). Marimastat, a specific inhibitor of MMPs (Peterson, 2006), inhibited 50% of the MMP activity at a concentration of 4.5 µM. Fluorescence measurements were recorded on a Shimadzu RF- 5301PC spectrofluorophotometer (Tokyo, Japan) at 328 nm (excitation) and 393 nm (emission). 7-Amino-4-methylcoumarin (MCA, Sigma-Aldrich, St. Louis, MO, USA) served as a standard. The results were expressed as µmol of MCA cleaved per min per g of protein in the homogenates.

### Morphology of liver tissue

#### Light microscopy

For morphological study of liver and heart tissue, samples were fixed with 10% neutral-buffered formalin. Specimens were embedded in paraffin and 5-µm cross-sections of tissues were stained with hematoxylin and eosin according to standard techniques. The slices were studied under light microscopy, using a STAR Carl Zeiss microscope (Jena, Germany).

#### Electron microscopic study

For the electron microscopic study of liver tissue, samples were first fixed in a mixture of 2% paraformaldehyde and 2.5% glutaraldehyde on 0.1 M phosphate buffer, postfixed in 1% osmium tetroxide solution, and then embedded in an Epon-Araldit mixture. Ultrathin tissue sections were obtained using an Ultramicrotome LKB-8800-V (Bromma, Vallingby, Sweden). Ultrastructural changes of liver cells were investigated with an electron microscope, JEM 1400 (Jeol, Japan).

### Statistical analysis

Data were analyzed by one-factor analysis of variance (ANOVA) and expressed as mean ± SEM. The difference between mean values was considered statistically significant at *p<*0.05. Analysis of the differences in the mean values between the groups (cysteine, aspartate proteases and MMP activity) were calculated using the non-parametric Kruskal-Wallis test, and statistically significant differences (*p<*0.05) were determined using the statistical program STATISTICA 10.0.

## Results

### Effect of repeated P-407 administration on liver, spleen, and heart weight

As compared to control mice receiving saline, mice repetitively administered P-407 had a significant (*p<*0.05) increase in body mass (30.1±0.35 g, *n*=23 *vs*. control 28.7±0.30 g, *n=*10). Repetitive administration of P-407 resulted in a significant increase in the relative weight of the liver at 24 h (5.4±0.14 g, *n*=15, *p<*0.001) and 5 days (4.6±0.13 g, *n*=10, *p<*0.001) following discontinuation of P-407 administration *versus* control (3.5±0.05 g, n=15). There were no changes in the relative heart weight (data not shown). Simultaneously, at the same time points, there was a significant (*p<*0.001) increase in the relative weights of spleen at 24 h (0.77±0.04 g/100 g of body weight) and 5 days (0.75±0.044 g/100 g of body weight) following the last dose of P-407 *versus* control (0.56±0.033 g/100 g of body weight).

### Effect of repeated P-407 administration on total serum cholesterol, HDL-cholesterol, non-HDL-cholesterol and TG concentrations

**Figure 1:**
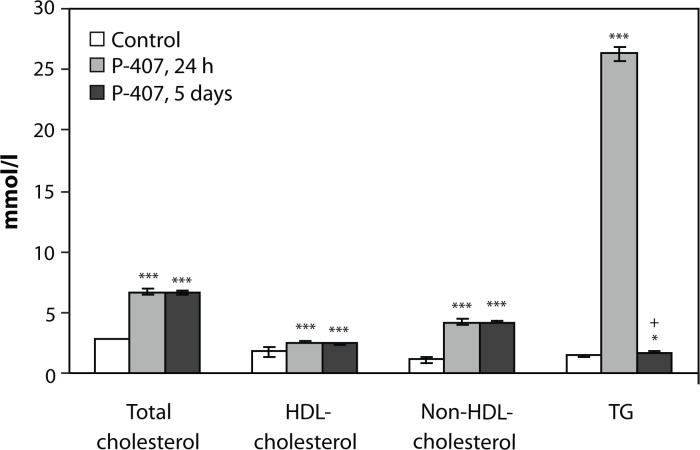
Influence of P-407 repeated administration in mice on serum total cholesterol, HDL-cholesterol, non-HDL-cholesterol and triglyceride concentration. X-axis – groups of animals (control and P-407-treated, 24 h and 5 days after the last P-407 injection); the bars left to right represent: Total cholesterol, HDL-cholesterol; non-HDL-cholesterol; TG. Y-axis – concentrations of total cholesterol, HDL-cholesterol, non-HDL-cholesterol and TG, mmol/L. The data are shown as mean ± SEM. Control group n=15, P-407-treated groups (n=15) at each point. Statistical significance is represented by: *p<0.05; ***p<0.001 vs. control; †*p<*0.001 – vs. P-407, 24 h. Here, as well as in Figures 2–5, P-407 was administered to mice as an intraperitoneal (i.p.) injection, at a dose of 500 mg/kg twice per week over the course of one month according to Johnston (2004). The animals were assessed in the experiment 24 h and 5 days after the last dose of P-407 or saline solution (in the control group).

Repetitive P-407 administration in mice induced increase in the total serum cholesterol at 24 h (*p<*0.001) and at the fifth day post-treatment (*p<*0.001) as well as total TG at 24 h, (*p<*0.001) and 5 days (*p<*0.05) post-treatment,relative to controls (Figure 1). Additionally, a significant increase of non-HDL-cholesterol and HDL-cholesterol was shown both at 24 h (*p<*0.001) and 5 days (*p<*0.001) following the last dose of P-407 (Figure 1). This increase in serum total cholesterol and TG was significantly greater than the rise in serum lipids observed after a single 500-mg/kg dose of P-407 observed earlier. With repeated P-407 administration, the serum concentration of TG 5 days after the last dose of P-407 decreased sharply (*p<*0.001, Figure 1) relative to the 24 h TG serum concentration; however it was still increased compared to control (*p<*0.05, Figure 1). These changes in serum TG were similar to the results following a single dose of P-407 (500 mg/kg) in mice reported previously.

### Plus-maze method of behavioral analysis of mice

**Figure 2:**
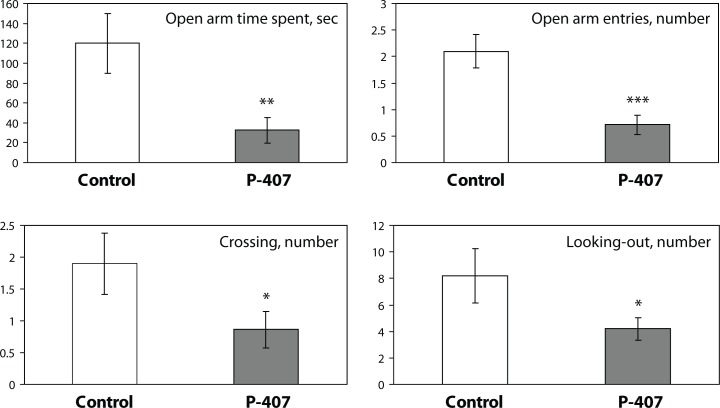
Effect of repeated P-407 administration on anxiety (elevated plus-maze test) in mice (Time spent on open arm, sec; Number of open arm entries; Number of crossings; Number of looking out the enclosed arm). The data are presented as mean ± SEM. Statistical significance is represented by: *p=0.05; **p=0.01; ***p=0.001 vs. control. The number of mice in the control group is 10, in repeated P-407 administration, 5 days, n=14. Each test duration is 5 min.

The behavior test was conducted in mice after repetitive administration of either saline (control, group 1, n=10) or P-407 (model, group 2, n=14). According to the plus-maze test, mice receiving P-407 were more anxious than controls (Figure 2). This was based on the following behavioral indices: reduced time spent on open arms (F_1,22_=8.68; *p=*0.01), a decrease in the number of open arm entries (F_1,22_=15.64; *p=*0.001), and a tendency to decrease in the number of looks out from the enclosed arm (F_1,22_=4.03; *p=*0.05) (Figure 2).

Simultaneously, we evaluated the motor activity of mice in the plus-maze test. It was demonstrated that the motor activity of P-407-treated mice also had a tendency to decrease. A decrease in motor activity was based on a lower number of movements from one enclosed arm to another (F_1,22_=3.81; p=0.05) (Figure 2). There was no change in the defecation level (data not shown).

### Effect of repeated P-407 administration on the specific activity of cysteine and aspartate proteases in heart and liver

Repetitive administration of P-407 resulted in a significant (*p<*0.01) increase in the specific activity of cathepsin B and cathepsin L in liver tissue relative to the control at both 24 h and 5 days following the last P-407 injection (Table 1). In heart, the specific activity of cathepsin B and cathepsin L was shown to increase significantly (*p<*0.01) at the 24 h time point when compared to controls (Table 1).

**Table 1:** Influence of poloxamer 407 administration in mice on cysteine and aspartate proteases specific activity in liver and heart.

Index	Control	P-407, 24 h	P-407, 5 days
**LIVER**			
Cathepsin B	0.66±0.052	0.93±0.127**	0.88±0.134**
Cathepsin L	0.41±0.011	0.66±0.059**	0.57±0.004**
Cathepsin D	0.04±0.002	0.05±0.004*	0.05±0.007
**HEART**			
Cathepsin B	0.11±0.011	0.20±0.024**	0.16±0.018
Cathepsin L	0.06±0.009	0.08±0.007**	0.06±0.003
Cathepsin D	0.02±0.010	0.08±0.020**	0.19±0.030***

P-407-treated mice, 24 h and 5 days after the last P-407 injection were used. Control group n=10, and P-407-treated groups n=11 at each point. Data are presented as mean ± SEM. Cathepsin B and cathepsin L specific activities are expressed in µmol MCA/min per g of protein, where MCA – 4-methylcoumarylamide; cathepsin D specific activity – in A366/min per mg of protein. Statistical significance is represented by:

* *p<*0.05 vs control.

** *p<*0.01 vs control.

*** *p<*0.001 vs control.

A significant increase in the specific activity of cathepsin D in the heart tissue of P-407-treated mice was shown 24 h after (*p<*0.01), and especially, 5 days after (*p<*0.001) the last P-407 injection (Table 1). In liver tissue, there was only a slight increase (*p<*0.05) in cathepsin D activity 24 h after (Table 1).

### Effect of repeated P-407 administration on MMP activity in heart and liver

**Figure 3:**
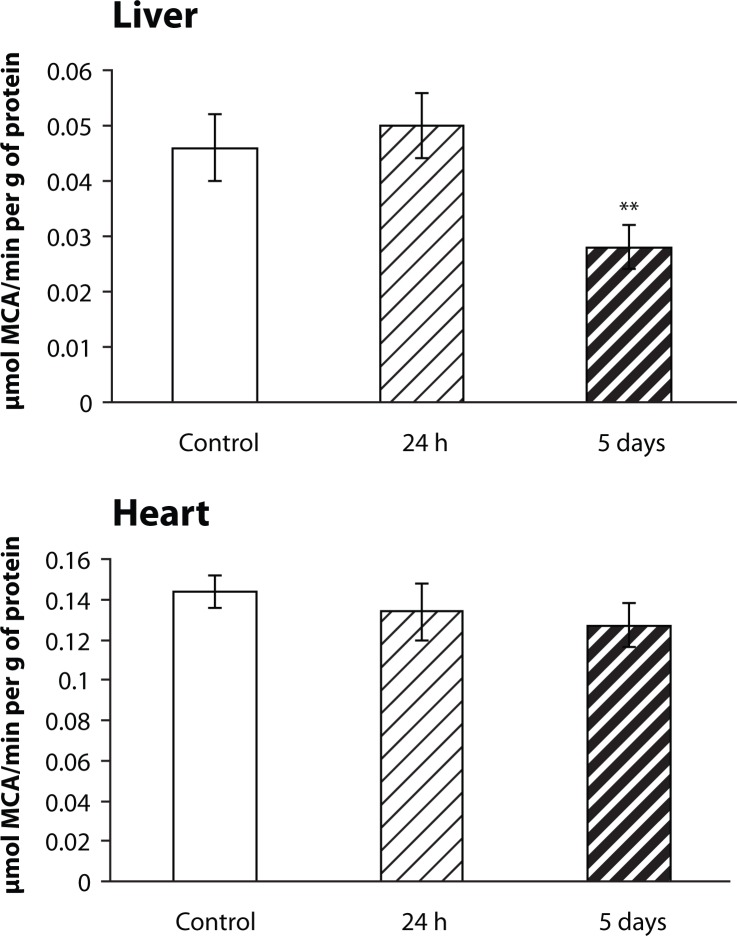
Effect of repeated P-407 administration on matrix metalloprotease (MMP) activity in liver and heart of mice. X-axis: Groups of animals. Control and P-407-treated, 24 h and 5 days after the last P-407 injection; Y-axis: general peptide substrate cleavage for MMP activity assay (MCA-Pro-Leu-Gly∼Leu-Dpa-Ala-Arg-NH_2_) cleaved activity, µmol MCA/min per g of protein. Number of animals in each group is 11. The data are shown as mean ± SEM. Statistical significance is represented by: ** *p<*0.01 vs control.

Following repetitive P-407 dosing, the activity of MMP was significantly (*p<*0.01) decreased in liver tissue on the 5^th^ day following the last dose of P-407 (Figure 3). There were no changes in heart MMP activity in P-407-treated mice at either time point when compared to controls (Figure 3), nor in serum MMP activity (data not shown). However, MMP activity in P-407-treated mice was significantly (*p<*0.001) decreased in the spleen at the 24 h (0.072±0.007, n=11) and the 5^th^ day post-P-407 treatment time points (0.089±0.014, n=11) relative to controls (0.129±0.013 µmol MCA/min per g of protein, n=11). It should be mentioned that the spleen is an organ known to be enriched with macrophages. Thus, one could speculate that spleen macrophages, like liver and lung macrophages, may potentially incorporate both P-407 and lipids by endocytosis.

### Morphology of liver and heart cells

#### Light Microscopy

**Figure 4:**
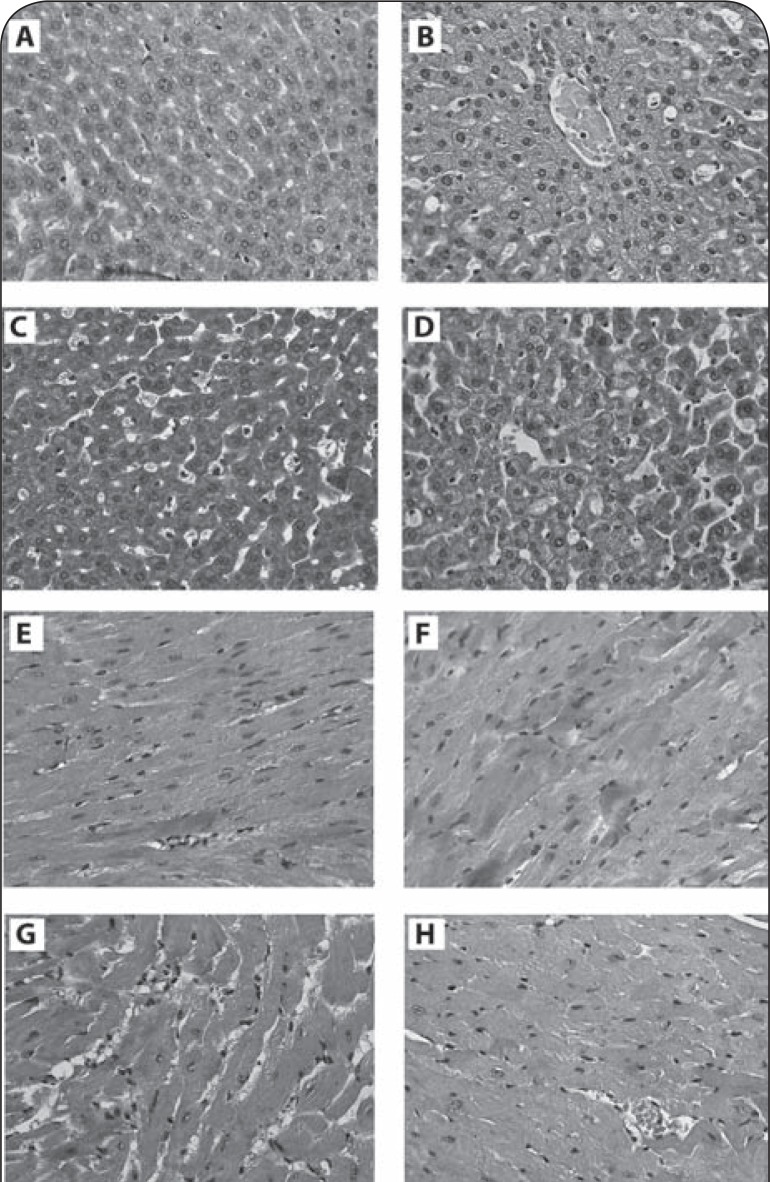
Influence of P-407 repeated administrations in mice on morphology of liver and heart cells (light microscopic study). **A–D**: Liver cells of mice. Magnification X 400. Hematoxilin-eosin staining. **A** – Liver cells of control mice; **B** – P-407, 24 h, Liver cells of mice with repeated P-407 administration. Signs of intrahepatic cholestasis; **C** – P-407, 24 h. Liver cells, venous stasis and discomplexation of liver cells; **D** – P-407, 5 days. Liver cells. Enlarged sinusoid, venous stasis and “net” appearance of liver cells. **E–H**: Heart cells of mice. Magnification ×400. Hematoxilin-eosin staining. **E** – control; **F** – P-407, 24 h; Heart tissue of mice with local changes of cardiomyocytes of contractile type; **G** – P-407, 24 h; Cardiomyocytes, interstitial edema; **H** –P-407, 5 days. Heart tissue of mice without significant changes (similar to control mice).

Analysis of liver cells obtained from mice 24 h after discontinuation of P-407 revealed signs of local intrahepatic cholestasis (Figure 4B) and venous stasis and discomplexation of liver cells (Figures 4C, 4D) relative to control tissue (Figure 4A). Five days after discontinuation of P-407 treatment there was evidence of increased cholestasis. In fact, an increase in the size of the sinusoids was noted (Figures 4D, 4E). Thus liver injury in P-407-treated mice was characterized by increased cholestasis and discomplexation of liver cells.

Changes observed in most cardiomyocytes of P-407-treated mice consisted primarily of damage to contractile-type cells (Figures 4F, 4G, 4H) when compared to controls (Figure 4E).

#### Electron Microscopy

**Figure 5:**
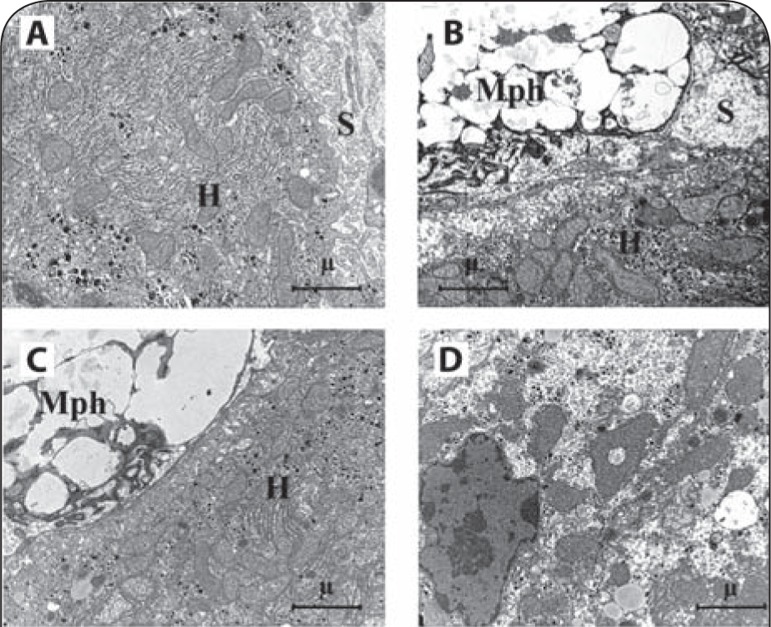
Influence of repeated P-407 administrations in mice on ultrastructure of liver (electron microscopy). **A** – Electron micrograph of liver cells (control). **B** – Electron micrograph of liver of mice with repeated P-407 administration after 24 h. In sinusoid lumen a large macrophage, filled by electron light granules. **C** – Liver cells in P-407-treated mice, 24 h. Macrophage overloaded by undigested material. **D** – Liver cell in P-407-treated mice, 24 h. Hepatocyte with “empty” zones in cytoplasma without subcellular structures. *Denotes*: Mph: macrophage, H: hepatocyte, S: sinusoid.

Electron microscopy (EM) studies of liver in P-407-treated mice were similar to previously reported EM findings using a single dose (500 mg/kg) of P-407 (Warren *et al*., 2011). Liver sinusoids were considerably expanded with dramatically enlarged macrophages containing granular material (Figure 5B) when compared to control liver (Figure 5A). The cytoplasm of macrophages obtained from the liver of P-407-treated mice exhibited a foamy appearance (Figure 5B) relative to controls (Figure 5A). The primary differences observed in cellular and tissue characteristics between mice receiving single and repeated P-407 administration included development of zones in hepatocytes without subcellular organelles (Figure 5C), as well as cytolysis of single hepatocytes, in mice. The cytoplasm of hepatocytes obtained from mice repeatedly administered P-407 exhibited numerous large vacuoles which contained electron-light material (Figure 5D).

## Discussion

The present investigation demonstrated that repeated (for one month) administration of P-407 to mice induced significant changes in the plasma lipid profile as well as biochemical and morphological changes in liver and heart. These changes include, but are not limited to, a drastic increase in serum atherogenic lipids, appearance of numerous lipid-laden macrophages, liver cholestasis, and overall behavioral changes. P-407-induced hyperlipidemia following single and repeated P-407 administration revealed many similarities, such as elevation in the total serum cholesterol, non-HDL-cholesterol, and TG. Similar findings were reported earlier by Johnston *et al*. (2001) following an identical single dose of P-407. Surprisingly, serum HDL was increased, albeit to a lesser extent. Elevation of serum HDL may possibly result from inhibition of endothelial lipase (Tanaka *et al*., 2009).

Heart and liver damage occurred in present study following administration of P-407 to mice for one month. Morphological heart damage, typical for atherosclerosis (including atherocalcinosis) occurred to a greater extent when P-407 was administered for a total of four months (Korolenko *et al*., 2012b). Restoration of serum lipids to baseline values does not occur by 5 days following discontinuation of P-407 administration for 1 month, as demonstrated earlier, when P-407 was administered as a single dose. In the present study, we also observed changes in animal behavior, such as increased anxiety, when P-407 was administered during a 1-month period. Similar changes were also noted in the 4-month model (unpublished data). An increase in anxiety has been reported in humans with atherosclerosis (Albert *et al*., 2005). In the present investigation, we found both a decrease in motor activity and an increase in relative body weight of the animals. Decreased motor activity possibly occurred as a result of significant lipemia and early development of atherosclerosis. Similar findings have been shown in older humans with atherosclerosis. In such cases, in regular exercise was recommended to prevent the development of atherosclerosis.

A difference in tissue morphology was observed between the single-dose and repeated-dose (1 month) P-407-induced models of hyperlipidemia and atherosclerosis in the present study. Interestingly, the appearance of organelle-free zones and cytolysis of some hepatocytes occurred in the 1-month model, as shown in the EM study, but this was not observed after a single dose of P-407 (Korolenko *et al*., 2012a). However, a common macrophage storage syndrome was observed in both models. Additionally, intracellular cholestasis was noted in models employing repeated P-407 administration for either 1 month (Figure 4) or 4 months. Intracellular cholestasis may result from impaired bile secretion, as suggested previously.

A significant difference in morphological and biochemical changes in liver and heart were observed between the two models in which mice received P-407 for either 1-month or 4 months. With the 1-month model, which replicates the early stages of atherosclerosis, there were morphological changes observed only in cardiomyocytes of the contractile type. This change is similar to ischemia of the heart, and was relatively mild when compared to mice used in the 4-months model. However, with the 4-month model, in which atherosclerosis is firmly established, there was dramatic damage of endothelial cells and atherocalcinosis (Korolenko *et al*., 2012b).

In the present study, liver tissue from control mice was shown to have a significantly greater specific activity of the lysosomal enzymes cathepsin B, cathepsin L, and cathepsin D, relative to heart tissue. This finding can probably be attributed to the low number of lysosomes in cardiomyocytes as compared to macrophages. In fact, the specific activity of cathepsin D and cathepsin B are known to be several times higher in macrophages when compared to hepatocytes (Lutgens *et al*., 2007). A significant increase in protease activity (except MMP) was demonstrated in liver and heart in the 1-month model of atherosclerosis induced by P-407 administration (early onset atherosclerosis), as well as with the 4-month-model in which atherosclerosis is more definitively established. However, with the 4-month model, the activity of lysosomal cathepsin B, cathepsin L, and especially cathepsin D, as well as MMP activity, increased several-fold in heart tissue (Korolenko *et al*., 2012b). These changes were observed simultaneously with damage to vascular endothelium in the heart.

In liver tissue, the specific activity of cathepsin B, cathepsin L, and cathepsin D was significantly increased 1 month after P-407 administration and attained even greater levels at 4 months (Korolenko *et al*., 2012b). There was no return of protease activity 5 days after discontinuing P-407 administration (Table 1). This fact may possibly reflect the steady overloading of liver macrophages with lipids and/or P-407 during this 1-month treatment period.

Lipid accumulation in macrophages was previously observed in the P-407-induced mouse model of atherosclerosis (Johnston, 2010). Macrophage storage syndrome (with lipids and/or P-407) can induce disturbances in lysosomal function of these cells, which may potentially play an important role in the pathogenesis of atherosclerotic foam cell formation (Lutgens *et al*., 2007; Qin & Shi, 2011). According to the literature, an increase in cathepsin B activity was shown *in vivo* and *in vitro* during incubation of hyperlipidemic postprandial serum with monocyte-derived macrophages (Jaffer *et al*., 2008). It has thus been suggested that an inadequate response of enzyme activity of lysosomal enzymes due to lipid overload may contribute to the development of atherosclerotic foam cells. These results also indicate that lipid accumulation blunts autophagic proteolysis *via* impairment of autophagosomal acidification and cathepsin expression (Inami *et al*., 2011).

Hepatic steatosis has recently been associated with a lysosomal pathway of apoptosis (Baskin-Bey *et al*., 2005; Ji *et al*., 2011) indicating that lysosomal permeabilization has occurred. Hepatocyte apoptosis and liver damage were reduced in Ctsb(–/–) and cathepsin B inhibitor-treated mice. These findings support a prominent role for the lysosomal pathway of apoptosis in steatotic livers following injury. The liver damage induced by administration of P-407 partially resembles hepatic steatosis and may represent a potential animal model for this disorder. However, future studies are required to support this premise.

In the present investigation, we have found a significant increase in cathepsin L activity in heart tissue. However, the biological role of cathepsin L is contrary to that of the cysteine protease cathepsin B (Brix *et al*., 2008). Cathepsin L is mainly located in the endosomal/lysosomal compartment, but a fraction of the proenzyme can be secreted and activated by other proteases, such as MMPs (Sun *et al*., 2011). Activated extracellular cathepsin L is capable of processing extracellular matrix (ECM) proteins, such as fibronectin, laminin, and type I, IV, and XVIII collagen, even at neutral pH (Reiser *et al*., 2010). Cathepsin L has been shown to participate in the remodelling of various tissues, including post-infarction cardiac tissue (Sun *et al*., 2011). Experimental myocardial infarction has been induced in Ctsl(–/–) mice, with a decreased survival rate by day 28 when compared to Ctsl(+/+) mice. These data indicate that cathepsin L regulates cardiac repair and remodelling after post-myocardial infarction through a mechanism with multiple pathways.

According to a recent study (Vasiljeva *et al*., 2007; Bromme & Wilson, 2011), cathepsins are necessary for cell survival and disruption of the regulation of the activity of these enzymes can cause serious diseases including atherosclerosis, Alzheimer's disease, and cancer. The design of cathepsin inhibitors may thus represent an important therapeutic intervention for these serious diseases (Cudic & Fields, 2009; Muller *et al*., 2012).

In conclusion, repeated administration of P-407 to mice for 1 month resulted in development of a mouse model of early “subclinical” cardiovascular disease (early atherosclerosis). Associated with early atherosclerotic changes were an increased serum level of total cholesterol, TG, atherogenic non-HDL-cholesterol, and increased anxiety level. The dyslipidemic phenotype resembled that which occurs in mice after a single injection of P-407. However, discontinuation of P-407 administration over 1 month does not cause lipids to return to baseline values within 5 days as previously observed following a single dose. Light and electron microscopy revealed changes of liver cells (cholestasis) and significant macrophage overloading. However in heart tissue, only contractile-type changes were noted. Additionally, the behavioral study demonstrated increased anxiety levels and a decrease in overall motor activity. There was also an increase in the activity of cysteine proteases (cathepsin B and cathepsin L) in heart and liver, and especially, of aspartate protease cathepsin D in heart tissue without significant changes in MMP activity. The increase of protease activity in cardiac tissue may suggest their early involvement in atherosclerosis following injury to cardiac cells. It is suggested that heart and liver cysteine and aspartate proteases may represent potential therapeutic targets in the early stages of atherosclerosis.
